# The protective effect of lipid emulsion in preventing bupivacaine-induced mitochondrial injury and apoptosis of H9C2 cardiomyocytes

**DOI:** 10.1080/10717544.2016.1261379

**Published:** 2017-02-06

**Authors:** Zhe Chen, Zhousheng Jin, Yun Xia, Shishi Zhao, Xuzhong Xu, Thomas J. Papadimos, Quanguang Wang

**Affiliations:** 1 Department of Anesthesiology, The First Affiliated Hospital, Wenzhou Medical University, Wenzhou, China,; 2 Department of Anesthesiology, The Ohio State University Medical Center, Columbus, OH, USA, and; 3 Department of Anesthesiology, University of Toledo College of Medicine and Life Sciences, Toledo, OH, USA

**Keywords:** Bupivacaine, mitochondria, apotosis, cardiomyocytes, lipid emulsion

## Abstract

Lipid emulsion (LE) has been shown to be effective in the resuscitation of bupivacaine-induced cardiac arrest, but the precise mechanism of this action has not been fully elucidated. Pursuant to this lack of information on the mechanism in which LE protects the myocardium during bupivacaine-induced toxicity, we explored mitochondrial function and cell apoptosis. H9C2 cardiomyocytes were used in study. Cells were randomly divided in different groups and were cultivated 6 h, 12 h, and 24 h. The mitochondria were extracted and mitochondrial ATP content was measured, as was mitochondrial membrane potential, the concentration of calcium ion (Ca2+), and the activity of Ca2+-ATP enzyme (Ca2+-ATPase). Cells from groups Bup1000, LE group, and Bup1000LE were collected to determine cell viability, cell apoptosis, and electron microscopy scanning of mitochondrial ultrastructure (after 24 h). We found that LE can reverse the inhibition of the mitochondrial function induced by bupivacaine, regulate the concentration of calcium ion in mitochondria, resulting in the protection of myocardial cells from toxicity induced by bupivacaine.

## Introduction

Bupivacaine, a long-acting amides local anesthetic, has been frequently used in regional block anesthesia. However, because of its cardiotoxicity, iatrogenic overdose or accidental injection into a vessel may induce circulatory collapse (Renehan et al., 2005). Lipid emulsions (LE) have been widely used in parenteral nutrition applications (Wretlind, 1972), but in 1998, Weinberg et al. identified Intralipid as an invaluable antidote in the resuscitatation of bupivacaine-induced cardiac arrest.

Bupivacaine inhibits carnitine metabolism in cardiac mitochondria (Weinberg et al., 2000). Some researchers have hypothesized that LE can reverse the inhibiting effect and restore fatty acid oxidation in mitochondria (Weinberg et al., 1997; Weinberg et al., 1998; Wong et al., 2010). Partownavid reported that fatty acid oxidation was to be indispensible for successful resuscitation of bupivacaine-induced cardiotoxicity. Eledjam et al. (1989) suggested that the inhibition of energy metabolism and proton transfer could be closely relevant to bupivacaine-induced cardiotoxicity. This was also supported by de La Coussaye et al. (1992). In 1998, Sztark et al. found that bupivacaine directly inhibited complex I of the mitochondrial respiratory chain, thereby impairing mitochondrial respiratory function and energy generation. Furthermore, bupivacaine has been shown to increase the proton permeability of the inner membrane of mitochondria and to decrease the mitochondrial membrane potential, which ultimately causes mitochondrial dysfunction (Heavner, 1986; Dabadie et al., 1987). In light of all this work, it is reasonable to pursue a line of questioning that bupivacaine may inhibit mitochondrial function. They also demonstrated that LE inhibits mitochondrial permeability transition pore (mPTP) opening, which might be responsible for its cardioprotective effects (Partownavid et al., 2012). Nonetheless, the effect of LE on bupivacaine-induced mitochondrial dysfunction is still unknown.

Thus, we hypothesized that LE would attenuate mitochondrial dysfunction induced by bupivacaine in cardiomyocytes by altering the regulation of Ca2 + concentration in mitochondria. In our study, we developed a research model of bupivacaine-induced cardiomyocyte toxicity, measured the effects of bupivacaine and LE on mitochondrial ATP content, membrane potential, Ca2+-ATPase activity, Ca2 + concentration, cell viability, apoptosis and mitochondrial ultrastructure.

## Materials and methods

### Materials

H9C2 cells were purchased from the Chinese Academy of Sciences (Shanghai, China). Additionally, the following were purchased: fetal bovine serum (FBS), pancreatic enzyme/EDTA, penicillin, and streptomycin from Gibco (Grand Island, NY); Dulbecco’s modified Eagle’s medium (DMEM) from Hyclone (Logan, UT); Annexin V-FITC from BD Biosciences (San Diego, CA); Fluo-3 AM from Life Technologies (Carlsbad, CA); and ATP assay kits and Adenosinetriphosphatase assay kits from NanJing JianCheng Bioengineering Institute (Nanjing, China). Cell Mitochondria Isolation Kits were purchased from Beyotime (Shanghai, China); bupivacaine and DiBAC4(3) from Sigma-Aldrich (St. Louis, MO); and 20% LE (Intralipid) from Sino-Swed Pharmaceutical Corp (Wuxi, China).

### Cell culture

H9C2 cells were cultured in high glucose DMEM medium with 10% FBS, 100 U/mL penicillin, and 100 μg/mL streptomycin at 37 °C in an atmosphere containing 5% CO_2_. The medium was renewed every 2 days. Bupivacaine hydrochloride was dissolved in dimethyl sulfoxide (DMSO) and medium, the needed concentrations were 100 μM and 1000 μM. The final concentration of DMSO was less than 0.5%. LE was adjusted to a final concentration of 1% using DMEM. When the cells occupied 80% of the dish bottom they were treated respectively with 100 μM bupivacaine (Group Bup100), 1000 μM bupivacaine (Group Bup1000), 1% LE (Group LE), 100 μM bupivacaine + 1% LE (Group Bup100LE), and 1000 μM bupivacaine + 1% LE (Group Bup1000LE).

### Cell viability assay

Cell viability was detected by Cell Counting Kit-8 (CCK8) assay (Agirre et al., 2015). H9C2 cells were collected and seeded at a density of 5 × 1000 cells/well in 96-well plates. Cell viability was determined at 24 h after treating with drugs according to the CCK8 assay kit. Briefly, 10 μL of CCK8 solution was added to 100 μL of the culture medium and optical density was measured at 450 nm wavelength after incubating for 2 h using a microplate reader (SpectraFluor, TECAN, Sunrise, Austria).

### Cell apoptosis assay

Cell apoptosis was detected by flow cytometry using the Annexin V-FITC/PI double-labeling method (Chen et al., 2016). Briefly, H9C2 cells were seeded in 24 well plates at a density of 5 × 10^5^ cells/well. After treating with 1 mM bupivacaine, 1% LE, or both drugs for 24 h, cells were collected and resuspended with 500 μL of Annexin V-FITC binding buffer according to the manufacturer’s instructions. Cells were then stained with Annexin V-FITC (5 μL) and PI (5 μL) for 10 min in the dark. Samples were analyzed by flow cytometry (BD Biosciences, Mountain View, CA).

### Isolation of mitochondria

Mitochondria were extracted by differential centrifugation (Li et al., 2013). H9C2 cells were collected 6, 12, and 24 h after treating with drugs by centrifugation at 600 *g*, 4 °C for 5 min; the supernatant was removed. Then 1 mL of mitochondrial isolation reagent was added into about 2 × 10^7^ cells, and the cells were homogenized after 10 min in an ice bath. The suspension was centrifuged at 600 *g*, 4 °C for 5 min, and the supernatant is transferred to another centrifuge tube, centrifuging at 11 000 *g*, 4 °C for 10 min. The precipitation was resuspended in mitochondrial storage solution.

### Mitochondrial membrane potentials assay

Mitochondrial membrane potential was detected using DiBAC4(3). Briefly, the mitochondria previously extracted were resuspended with 1 mL phosphate-buffered saline (PBS) and transferred to a 1.5 mL centrifuge tube. Then DiBAC4(3) was added to a final concentration was 0.5 μM. After incubating in the dark for 30 min at a temperature of 37 °C, the mitochondria were collected and washed with PBS twice. The precipitant was resuspended in 400 μL PBS and measured at 488 nm wavelength by flow cytometry. The emission wavelength was about 516 nm and the fluorescence was blue-green.

### Mitochondrial calcium ion detection

Mitochondrial calcium ion was detected using Fluo-3 AM. Briefly, the mitochondria were extracted in the steps mentioned before and resuspended with PBS. Fluo-3 AM was added to the mitochondria and the final concentration was 10 μM. After 1 h of incubation, the mitochondria were washed with PBS without calcium thrice. Samples were analyzed by flow cytometry. The fluorescence intensity of each sample was recorded as *F*. The *F*_max_ value was recorded as the fluorescence intensity when the mitochondria were incubated with 1% Triton X100 for 30 min and 1 mM calcium chloride for 10 min at room temperature. The *F*_min_ value was determined as the fluorescence intensity when the mitochondria was incubated with 10 mM EDTA for 5 min. The concentration of calcium ion was calculated using the formula (Kd = 390 nM):

$$\text{The concentration of Ca2}+\;=\mathrm{Kd}\times (F-F_{\min})/(F_{\max}-F).$$

### ATP content and calcium ATPase activity assay

Mitochondrial ATP content was analyzed by colorimetry using phosphomolybdic acid. Mitochondrial calcium ATPase activity was determined by the amount of phosphorus generated by ATPase and substrate.

### The ultrastructure of mitochondria

The ultrastructure of mitochondria was reported using electron microscopy. Briefly, H9C2 cells were collected after the intervention and fixed by glutaraldehyde. This was followed by osmic acid. Subsequently, specimens were dehydrated by acetone and then embedded. Thin and semi-thin specimen slices were made, and scanning electron microscopy was carried out to observe the cellular mitochondrial structure.

### Statistical analysis

Statistical analysis was done using SPSS 17.0. Values were expressed as the mean ± SD. Multiple comparisons between groups were analyzed by one-way ANOVA. Least significant difference (LSD) test was performed as post hoc analysis for multiple comparisons between groups. Dunnett’s T3 test was performed when the variance was irregular. *p* < 0.05 was considered to be statistically significant.

## Results

### Mitochondrial ATP content

**Figure 1.**
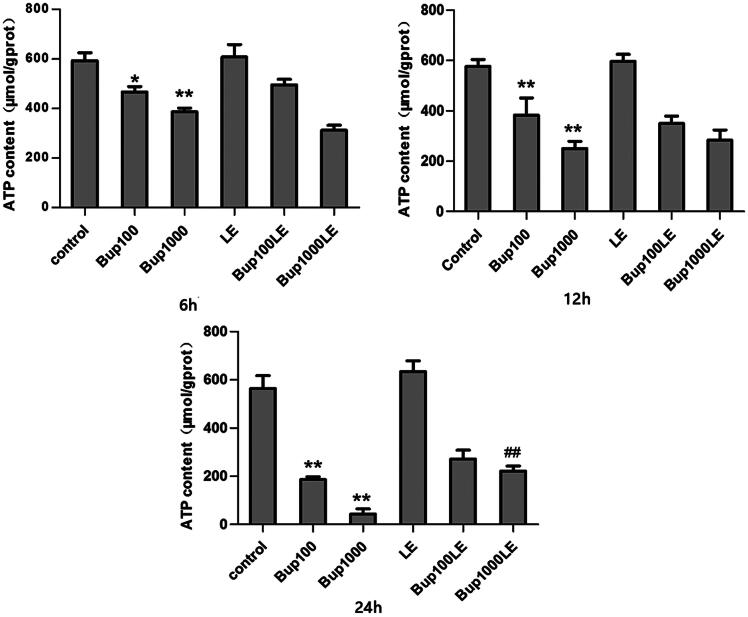
The content of mitochondrial ATP in each group after incubating for 6 h, 12 h, and 24 h. It was measured by commercial kits. Date represented are mean ± SD of 3 separate experiments. *p < 0.05, **p < 0.01 versus the control group. ##*p* < 0.01 versus group Bup1000.

Compared with the control group, the mitochondrial ATP content of cells in group Bup100 was decreased at 6 h (group Bup100 versus group control *p* = 0.010), and further reduced at both 12 h (group Bup100 versus group control *p* = 0.005) and 24 h (group Bup100 versus group control *p* = 0.001). Mitochondrial ATP content of cells in group Bup1000 was substantially reduced when compared with group Bup100 at 12 h (group Bup1000 versus group Bup100 *p* = 0.036) and 24 h (group Bup1000 versus group Bup100 *p* = 0.012). Mitochondrial ATP content of LE group had no statistical difference compared with the control group at any time. At 24 h, ATP content in group Bup1000LE was increased compared with Bup1000 group (group Bup1000LE versus group Bup1000 *p* = 0.003) (Figure 1).

### Calcium ATPase activity

**Figure 2.**
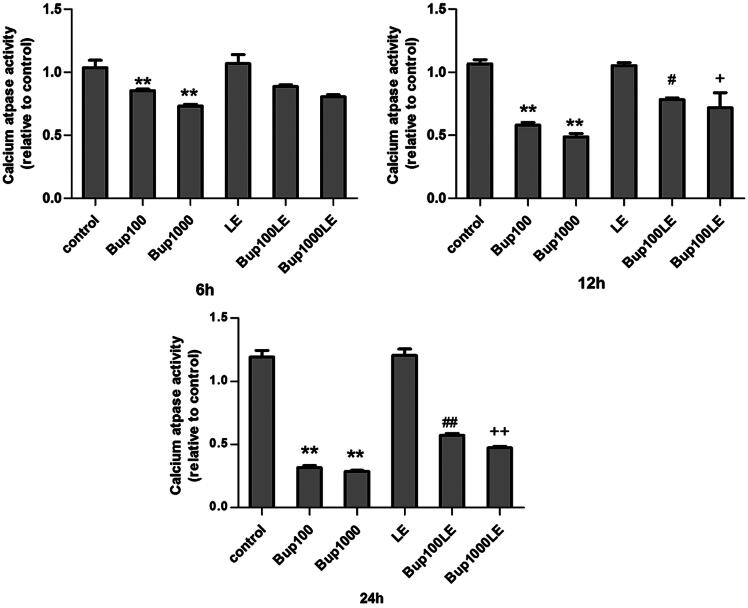
Mitochondrial calcium ATPase activity was measured by commercial kits after cells were incubated for 6 h, 12 h, and 24 h. Date represented are mean ± SD of 3 separate experiments. *p < 0.05, **p < 0.01 versus the control group. ##*p* < 0.01 versus group Bup100. ++*p* < 0.01 versus group Bup1000.

Mitochondrial calcium ATPase activity of group Bup100 was reduced at 6 h as compared with the control group (Bup100 versus group control *p* = 0.007), and was further reduced at 12 h (group Bup100 versus group control *p* < 0.001) and at 24 h (group Bup100 versus group control *p* < 0.001). Mitochondrial calcium ATPase activity of group Bup1000 was substantially reduced when compared with group Bup100 at 6 h (group Bup1000 versus group Bup100 *p* < 0.048), but demonstrated no statistical difference at 12 h and 24 h. The LE group and the control group were similar. However, calcium ATPase activity in groups Bup100LE and Bup1000LE improved significantly as compared to group Bup100 and group Bup1000 respectively at 12 h (group Bup100LE versus group Bup100 *p* = 0.021; group Bup1000LE versus group Bup1000 *p* = 0.011) and 24 h(group Bup100LE versus group Bup100 *p* < 0.001; group Bup1000LE versus group Bup1000 *p* = 0.001) (Figure 2).

### The mitochondrial membrane potential detection

**Figure 3.**
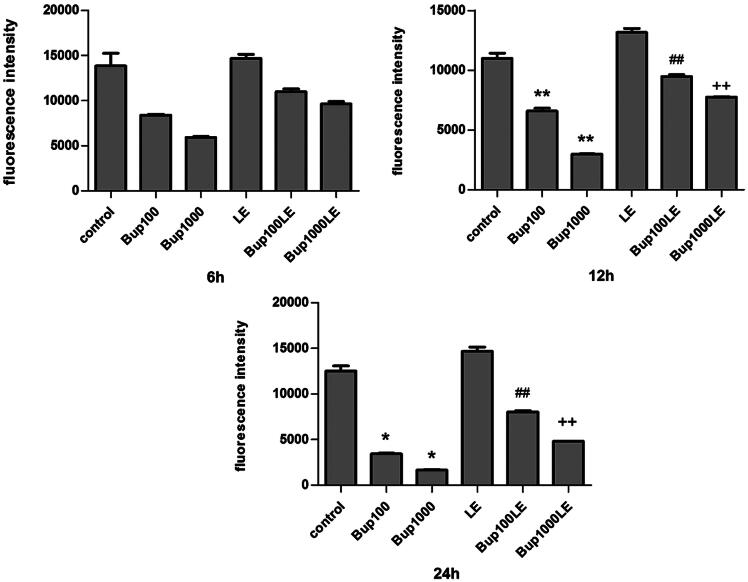
Mitochondrial membrane potential was measured using DiBAC4(3) and detected by flow cytometry. The fluorescence was blue-green and fluorescence intensity was detected after cells were incubated for 6 h, 12 h, and 24 h. Date represented are mean ± SD of 3 separate experiments. *p < 0.05, **p < 0.01 versus the control group. ##*p* < 0.01 versus group Bup100. ++*p* < 0.01 versus group Bup1000.

Compared with the control group, the mitochondrial membrane potential of groups Bup100 and Bup1000 began to decline at 6 h, but the results were not statistically significant. Whereas the results of these two groups had significant differences as compared with the control group at 12 h (group Bup100 versus group control *p* < 0.001; group Bup1000 versus group control *p* < 0.001) and at 24 h (group Bup100 versus group control *p* = 0.015; group Bup1000 versus group control *p* = 0.010). The mitochondrial membrane potential of LE group was no different than the control group, but a 1% LE improved the mitochondrial membrane potential disturbed by bupivacaine. As shown in Figure 3, the results of group Bup100LE and Bup1000LE were statistically significant compared with group Bup100 and group Bup1000 respectively at 12 h (group Bup100LE versus group Bup100 *p* < 0.001; group Bup1000LE versus group Bup1000 *p* < 0.001) and at 24 h (group Bup100LE versus group Bup100 *p* < 0.001; group Bup1000LE versus group Bup1000 *p* = 0.001) (Figure 3).

### Mitochondrial Ca2+

**Figure 4.**
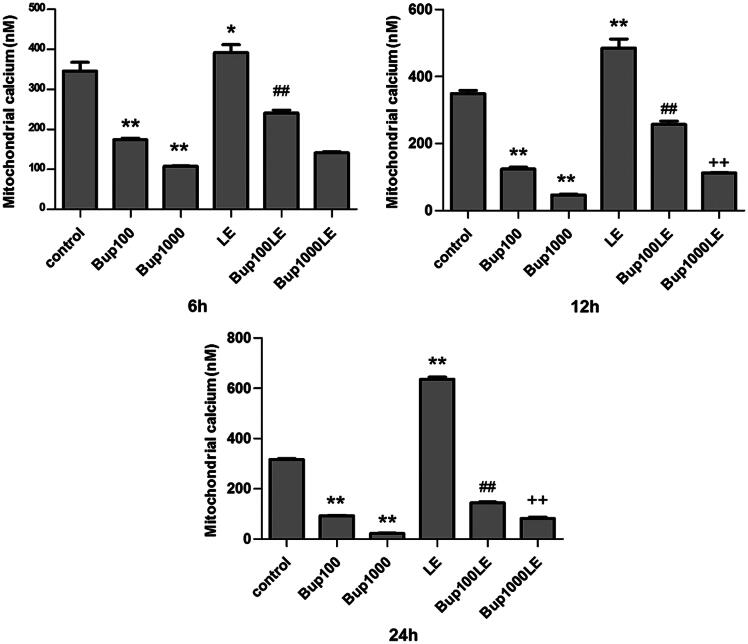
Mitochondrial calcium ion detection was detected by flow cytometry using Fluo-3 AM. The concentration of calcium ion was detected after cells were incubated for 6 h, 12 h, and 24 h. Date represented are mean ± SD of 3 separate experiments. *p < 0.05, **p < 0.01 versus the control group. ##*p* < 0.01 versus group Bup100. ++*p* < 0.01 versus group Bup1000.

The concentration of calcium ions of mitochondria in group Bup100 and group1000 were both reduced when compared to the control group at 6 h (group Bup100 versus group control *p* < 0.001; group Bup1000 versus group control *p* < 0.001), 12 h (group Bup100 versus group control *p* < 0.001; group Bup1000 versus group control *p* < 0.001) and 24 h (group Bup100 versus group control *p* < 0.001; group Bup1000 versus group control *p* < 0.001). The concentration of calcium ions of mitochondria in group LE was increase at 6 h (group LE versus group control *p *=* *0.025), and more so at 12 h (group LE versus group control *p *<* *0.001) and 24 h (group LE versus group control *p *<* *0.001). After 24 h, the results of group Bup100LE and Bup1000LE were statistically significant compared with group Bup100 and group Bup1000 respectively at 24 h (group Bup100LE versus group Bup100 *p* < 0.001; group Bup1000LE versus group Bup1000 *p* < 0.001) (Figure 4).

### Cell viability and apoptosis

**Figure 5.**
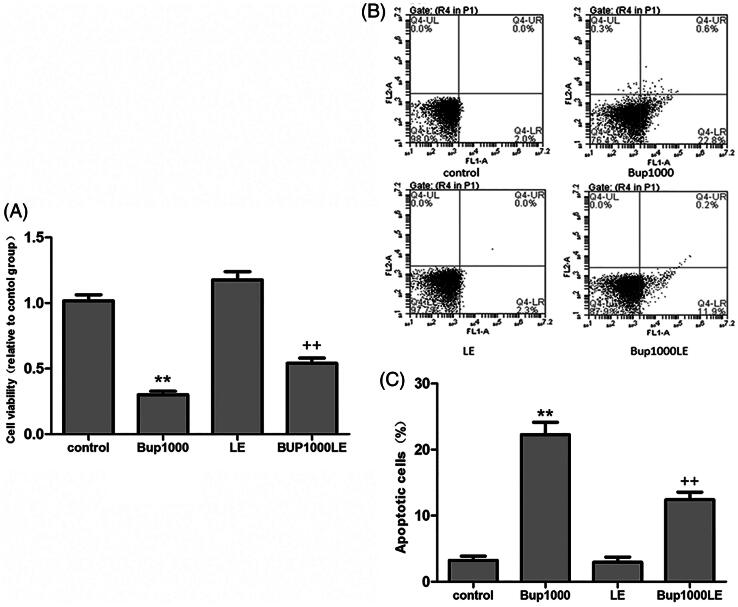
(A) Cell viability was detected by CCK8 assay after cells were incubated for 24 h. (B and C) Cell apoptosis was detected by flow cytometry using the Annexin V-FITC/PI double-labeling method. Date represented are mean ± SD of 3 separate experiments. **p < 0.01 versus the control group. ++*p* < 0.01 versus group Bup1000.

After 24 h, the cell viability in group Bup1000 decreased significantly as compared to the control group (group Bup1000 versus group control *p* < 0.001), and the result in group LE had no difference compared with group control. The cell vitality in Bup1000LE was significantly higher than group Bup1000 (Figure 1). After 24 h, the cell apoptosis in group Bup1000 were much more than group control, the difference was statistically significant (group Bup1000 versus group control *p* < 0.001), and 1% fat emulsion significantly reduce cell apoptosis caused by bupivacaine (group Bup1000LE versus group Bup1000 *p *<* *0.001) (Figure 5).

### Ultrastructure of mitochondria

**Figure 6.**
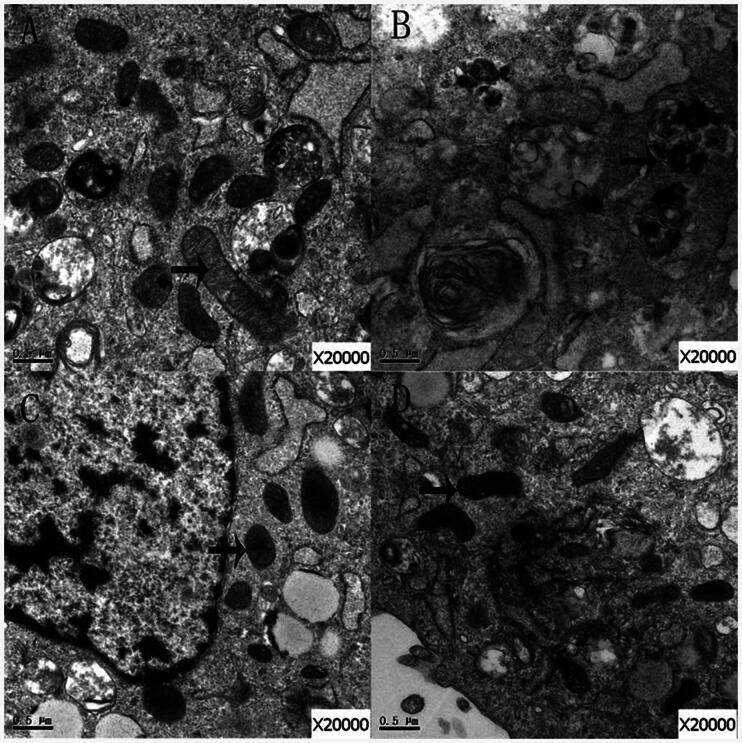
The mitochondria of cells in group control (A), group Bup1000 (B), group LE (C) and group Bup1000LE (D) were scanned by electron microscope. The arrow is mitochondria.

Mitochondria were scanned with electron microscopy. In the control group, the structures of the mitochondria were complete and the mitochondrial cristae were visible (Figure 6A). However, the structures of mitochondria in group Bup1000 were abnormal (Figure 6B), but the structures of mitochondria in group LE were intact (Figure 6C). In group Bup1000LE, the mitochondrial structure demonstrated morphological heterogeneity, and the mitochondrial cristae were swollen, but the general structure of the mitochondria were still intact (Figure 6D).

## Discussion

This study demonstrated that group Bup100 and group Bup1000 decreased mitochondrial ATP content, mitochondrial membrane potential, and significantly reduce mitochondrial Ca2 + in H9C2 cardiomyocytes. LE (1%) can reverse the bupivacaine-induced decrease in ATP content, the decrease in membrane potential and the decrease in Ca2 + concentration in mitochondria. Furthermore, LE improve the ultrastructure of mitochondria and reduce bupivacaine-induced apoptosis.

The results of our study indicate that 1000 μM bupivacaine applied to H9C2 cardiomyocytes over 24 h could reduce cell viability and increase the number of apoptotic cells. Yang et al. (2015) reported similar results under the same conditions; the ratio of necrotic apoptosis cells was 24.66 ± 2.57%, which was in consistent with our study. Thereby confirming that bupivacaine can lead to cardiomyocyte necrosis and apoptosis. At the same time, we found that 100 μM and 1000 μM bupivacaine significantly inhibited mitochondrial ATP production and this effect was profound at 24 h. Zhang et al. (2003) reported that bupivacaine could reduce the myocardial ATP content of heart tissue in a New Zealand rabbit model of local anesthetic-induced cardiac toxicity, and they suggested that it was related to bupivacaine induced inhibition of fatty acid oxidation. Weinberg et al. (2000) also reported that bupivacaine can inhibit the myocardial mitochondria carnitine transposition enzyme thereby inhibiting fatty acid oxidation and myocardial energy supplementation. Another study demonstrated that bupivacaine may cause mitochondrial oxidative phosphorylation uncoupling, inhibit mitochondrial aerobic respiration, and reduce the generation of cellular ATP (Sztark et al., 1998). This uncoupling effect of bupivacaine may alter the mitochondrial membrane potential and inhibit mitochondrial respiration, which would result in the generation of reactive oxygen species in the mitochondria, thus triggering mitochondrial apoptotic pathways (Perez-Castro et al., 2009; Cela et al., 2010). Our results suggest that bupivacaine toxicity, especially cardiac toxicity, is associated with inhibition of mitochondrial function which results in inefficient cardiomyocytes energy production.

Dabadie et al. (1987) showed that the lipid solubility of bupivacaine can uncouple rat liver mitochondria. They reported that lipid soluble local anesthetics cross the cytoplasmic membrane, accumulate, disturb the transmembrane potential, and uncouple oxidative phosphorylation and suppress mitochondrial metabolism. In our study, we found that the mitochondrial membrane potential decreased significantly in group Bup1000. Such an action result in the disappearance of an effective membrane potential thereby activating the mitochondrial apoptosis pathway. We found that a LE reverses H9C2 cardiomyocyte apoptosis caused by bupivacaine, as demonstrated by the decreased numbers of apoptotic cells in the mixture of LE and bupivacaine group as compared to the purely bupivacaine group. We also have demonstrated that there is less reduction of ATP content and the membrane potential in the mixed group (bupivacaine and LE). Our results suggest that LE can reduce apoptosis, and at the same time, it also can improve mitochondrial energy production and the stability of the membrane potential, which leads us to speculate that LE can remove the inhibition of mitochondrial function induced by bupivacaine, thereby reversing the toxicity of bupivacaine.

Intracellular calcium homeostasis is maintained by the mitochondria through the calcium ATP enzyme (calcium pump) action on the endoplasmic reticulum and the intracellular calcium pool. The concentration of free calcium in mitochondria is closely related to the proper functioning of mitochondria (McCormack & Denton, 1980; Cortassa et al., 2003). In a thermal dynamics model experiment, which addressed mitochondrial energy metabolism, Cortassa et al. (2003) reported that intramitochondrial free calcium ions can activate isocitric dehydrogenase and oxoglutarate dehydrogenase in the tricarboxylic acid cycle, and promote ATP generation. In our experiment, after 6 h after bupivacaine alone, the concentration of calcium ion in mitochondria decreased significantly. Also, extending the bupivacaine contact time increased the decline of calcium. This trend was consistent with the bupivacaine-induced decrease in mitochondrial ATP content and membrane potential, leading us to conclude that bupivacaine (alone) altered cell mitochondrial membrane structure in a detrimental fashion causing mitochondrial membrane permeability and mitochondrial calcium loss, which resulted in dysfunctional mitochondria. In normal cells, the calcium pump on the mitochondria membrane will transfer and store the calcium from the cytoplasm to the mitochondria in an inverse concentration gradient, thus maintaining a high calcium balance in the mitochondria and a low calcium balance in the cytoplasm. Our results demonstrate that the activity of the mitochondrial Ca2 + ATP enzyme in the bupivacaine group was inhibited significantly, and exacerbated the decrease of the calcium ion in the mitochondria. Partownavid et al. (2012) reported that LE can increase the buffering capacity of mitochondria in relation to calcium ions, thereby delaying the opening of the permeability transition pore of mitochondria resulting in the protective effect of the cell. Huang et al. (1992) also pointed out that LE could increase calcium channel activity on the plasma membrane, which could explain our results showing that free calcium ions were significantly increased with LE alone, and after mixing LE with bupivacaine.

There were limitations to this study. We did not use primary rat cardiomyocytes and we could not observe myocardial contractility. Additionally, the drug concentrations we used in our experiments did not allow us to observe a dose effect relationship between bupivacaine and LE on the mitochondrial indicators.

In conclusion, our results suggested that LE can reverse the inhibition of the mitochondrial function induced by bupivacaine, regulate the concentration of calcium ion in mitochondria, resulting in the protection of myocardial cells from toxicity induced by bupivacaine. These findings provide a new direction for the study of the mechanism of LE in the reversal of bupivacaine-induced cardiac toxicity.
